# Supplementary material for: Quasi-seamless stitching for large-area micropatterned surfaces enabled by Fourier spectral analysis of moiré patterns
Source: Nat Commun. 2023 Apr 18;14:2202. doi: 10.1038/s41467-023-37828-8 (PMC10113184; doi:10.1038/s41467-023-37828-8)
Supplement: Supplementary file 1 — Supplementary Information [file 41467_2023_37828_MOESM1_ESM.pdf]

Supplementary Information

Quasi-seamless stitching for large-area micropatterned surfaces enabled by Fourier spectral analysis of moiré patterns

Woo Young Kim[1], Bo Wook Seo[1], Sang Hoon Lee[1], Tae Gyung Lee[1], Sin Kwon[2], Won Seok Chang[3,4], Sang-Hoon Nam[5], Nicholas X. Fang[5,6], Seok Kim[1,7]*, Young Tae Cho[1,7]*

[1]Department of Smart Manufacturing Engineering, Changwon National University, South Korea

[2]Department of Flexible & Printed Electronics, Korea Institute of Machinery and Materials, South Korea

[3]Department of Nano Manufacturing Technology, Korea Institute of Machinery and Materials, South Korea

[4]Department of Nanomechatronics, University of Science and Technology, South Korea

[5]Department of Mechanical Engineering, Massachusetts Institute of Technology, United States

[6]Department of Mechanical Engineering, University of Hong Kong, Hong Kong

[7]Department of Mechanical Engineering, Changwon National University, South Korea

*Corresponding author: kimseok@changwon.ac.kr; ytcho@changwon.ac.kr

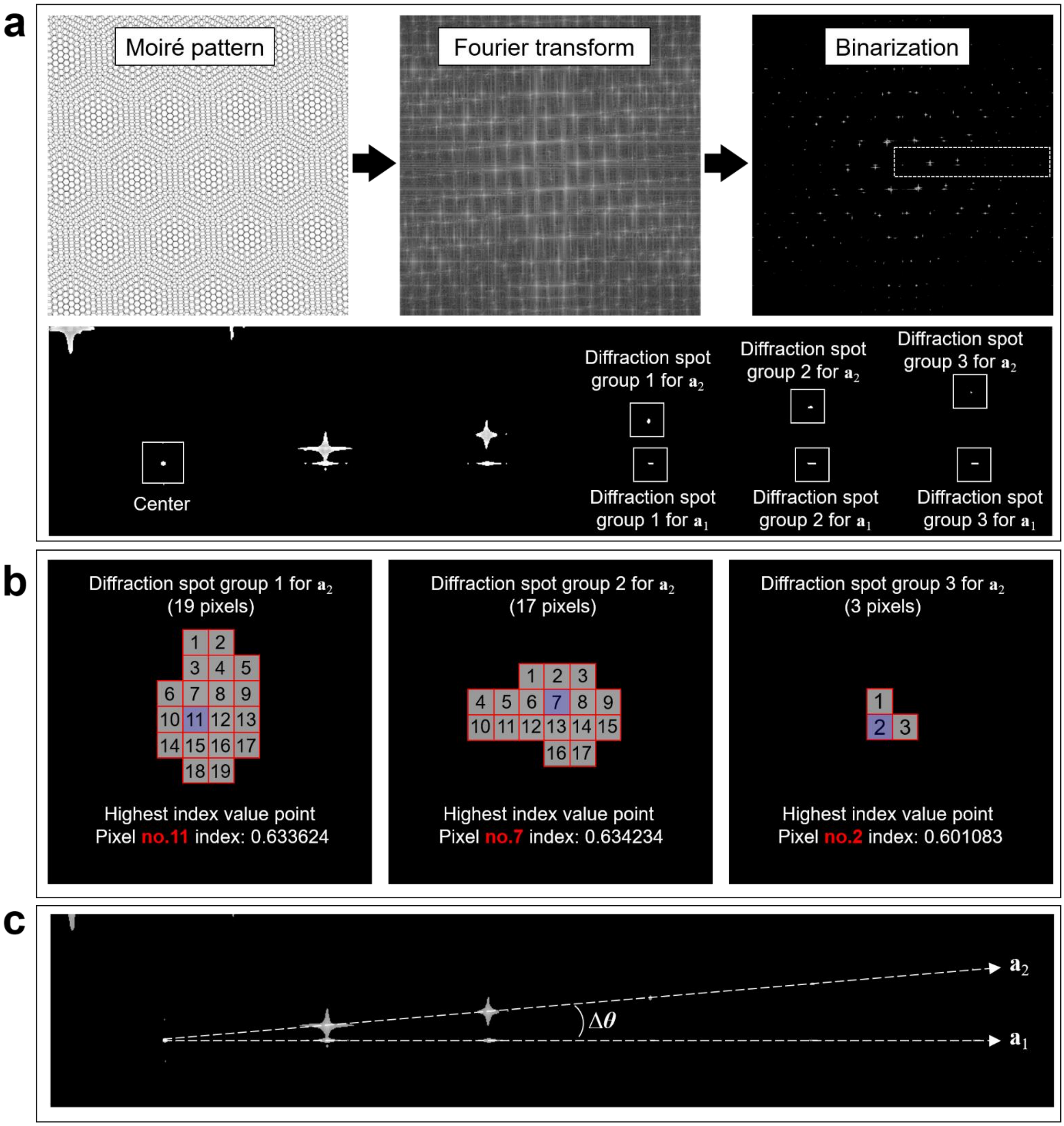

Supplementary Figure. 1 The calculation procedure for estimating for the rotation angle between the reciprocal lattice vectors in the Fourier transformed images. a Extraction of three diffraction spot groups farthest from the center position in the binarized image **b** Determination of the highest index value point from diffraction spot groups **c** Calculation of the rotational offset from the reciprocal lattice vectors of $\mathbf{a}_1$ and $\mathbf{a}_2$

The measured rotation angle is a linear function of the input rotation angle ($\theta_M = \theta_R$) based on the rotational invariant property of Fourier transform. To evaluate the estimation of rotational offset on our method, we analyzed the rotational offset for angles below 10º in **Fig. 2b** and **Supplementary Fig. 2b**, considering realistic conditions in actual applications. Here, the following is a detailed procedure to calculate the estimated rotation angle in the Fourier transformed images. The spectrum distribution of the Fourier transformed images can be influenced by various factors, including the degree of periodicity, complexity, and shapes of the constituent patterns, as well as the pixel resolution of the captured image by the vision system. These factors can significantly affect the accuracy of the calculation of the rotational offset. To minimize the uncertainty stemming from image pixel resolution, we utilized the three diffraction spot groups farthest from the center

position in the binarized images (**Supplementary Fig. 1a**). In each of these diffraction spot groups, the highest index values were selected as representative points, thus allowing to determine the reciprocal lattice vectors $\mathbf{a}_1$ and $\mathbf{a}_2$, as indicated in **Supplementary Fig. 1b**. By calculating of the angle between the reciprocal lattice vectors, we were able to estimate the rotational offset in the Fourier transformed images (**Supplementary Fig. 1c**), resulting in the corresponding rotational offset of the overlapped patterns in the real space due to the rotational invariant properties. This type of principle used for calculating the rotational angle in this work has been widely utilized to interpret the twist angle of bilayer graphene (BLG) as moiré patterns resulting from misorientations between graphene layers[1,2].

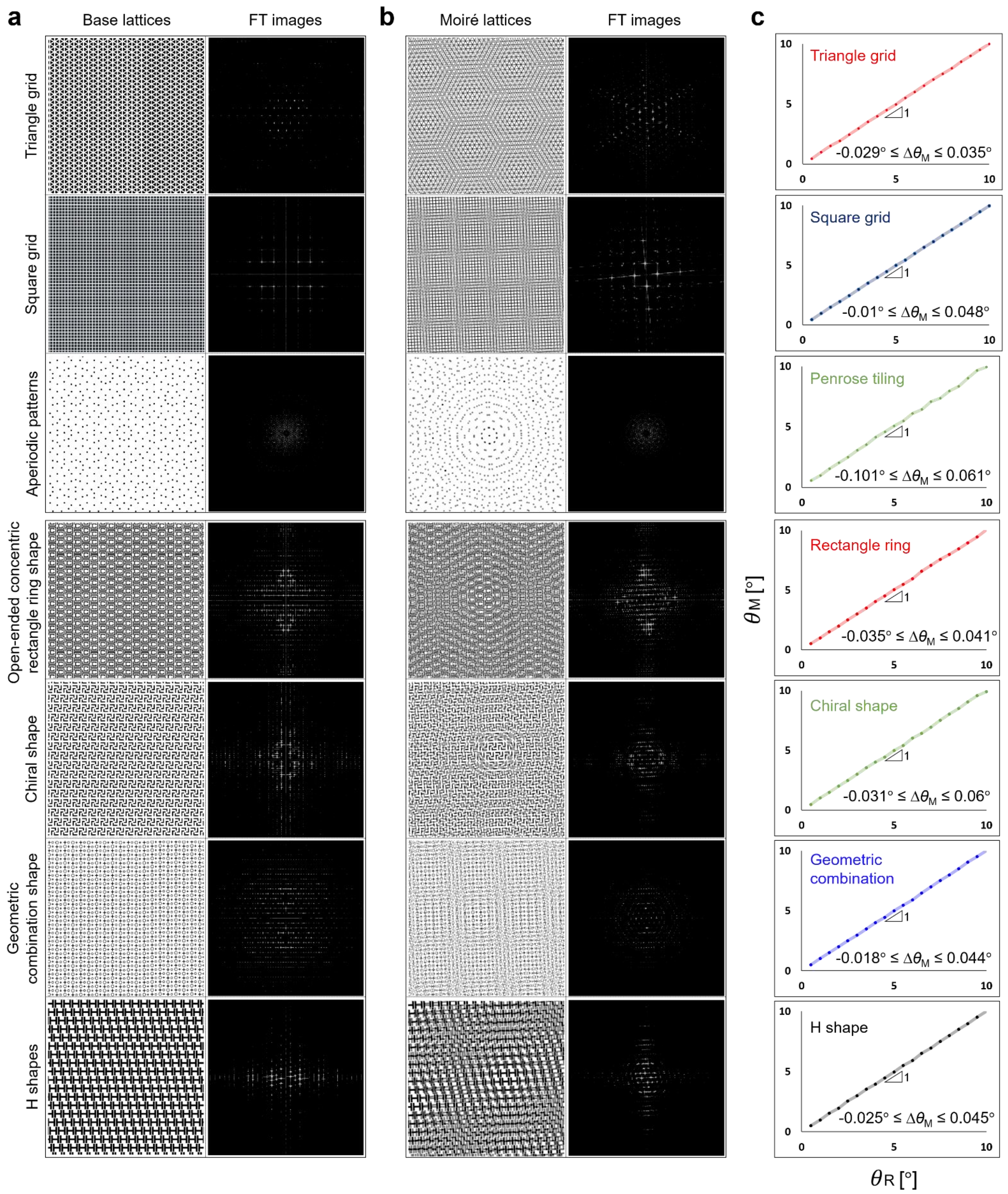

Supplementary Figure. 2 Simulated results for the extraction of the rotational offset using Fourier spectral analyses of the moiré lattices formed by two identical overlapped patterns. Simulated images and corresponding FTed images of **a** base and **b** moiré lattices of various complicated patterns such as aperiodic patterns of Penrose tiling and classical patterns for metasurfaces. **c** The measured rotation angle (θ_M) as a function of the input rotation angle (θ_R).

The uncertainty in estimating the rotational offset in overlapped patterns can be affected by various geometrical factors of the constituent patterns, such as the degree of periodicity, complexity, and shapes. As shown in **Supplementary Fig. 2**, we speculate that the slightly increased uncertainty observed in the aperiodic pattern of Penrose tiling may be attributed to the increased diffraction components of spatial frequency

spectrum resulting from the aperiodicity of the constituent patterns. Therefore, we also plan to implement a machine learning-based algorithm to improve the accuracy of estimating the rotational offset of overlapped patterns using the MAPI process[3].

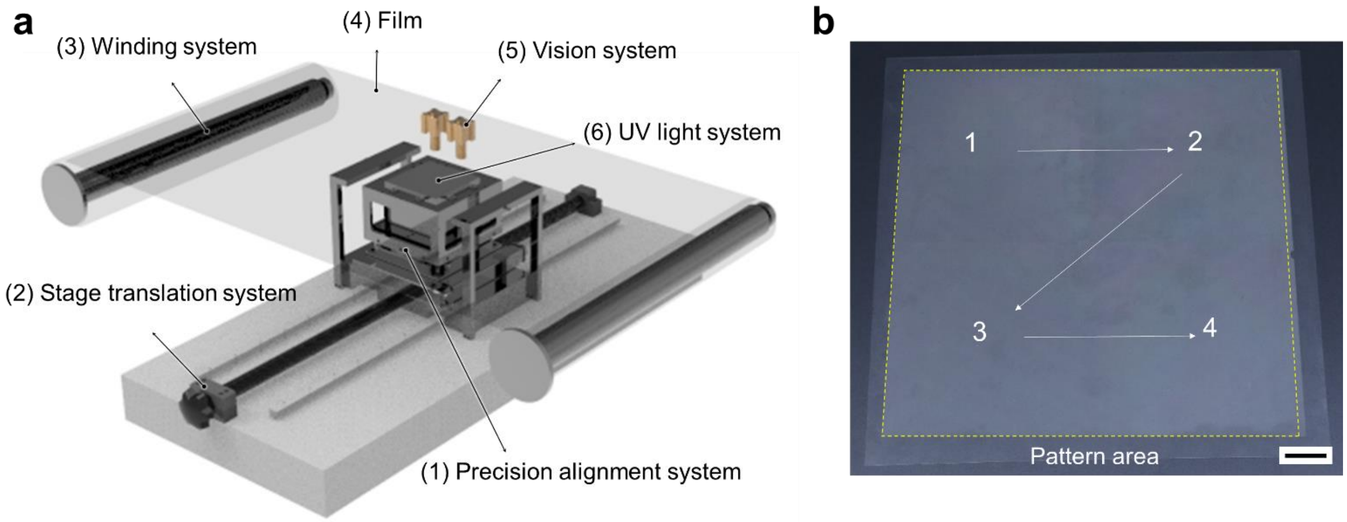

Supplementary Figure. 3 Systems for the MAPI process. a Schematic of the roll-to-plate printing system used to fabricate quasi-seamless large-area microstructured surfaces using the MAPI process, which was comprised of the precise motorized stages and vision system. **b** The representative printing result of large-area micropatterned surfaces (200 × 200 mm^2). Scale bar: 20 mm.

In the MAPI process (**Supplementary Fig. 3a**), the objective is to analyze the overlapped area between the imprinted pattern and MAPI stamp pattern using the vision system (5). After the initial UV NIL procedure, the stage is moved to the next patterning area using the stage translation system (2). The resin is then filled between the MAPI stamp and the film (4). The vision system (5) analyzes the overlapped pattern area in real-time to measure the rotational and translation offsets, and the MAPI stamp and film is precisely aligned (**Fig. 1b**) using the precision alignment system (1). The cured resin is demolded after applying UV exposure. The MAPI process is repeated to create large-area patterns (**Supplementary Fig. 3b**). In contrast, the manual stitching process only roughly aligns the patterns using the stage translation system (2) and the winding system (3) without precise alignment via the vision system (5).

In addition, the MAPI process involves the following steps:

(Step.1) capturing the moiré patterns in the overlapped patterns using the vision system
(Step.2) performing the Fast Fourier transform (FFT) of the captured moiré patterns to extract the bright node points in the *k*-space
(Step.3) binarizing the Fourier transformed images to clarify these specific node points
(Step.4) calculating the rotational offset in post-processed images
(Step.5) compensating for the rotational offset
(Step.6) capturing the overlapped patterns after compensating for the rotational offset
(Step.7) calculating the translation offsets using vision-based shifted pixel analyses
(Step.8) compensating for the translational offsets

The low computational load of the FFT algorithm and vision-based shifted pixel analysis enables efficient extraction of the rotational and translational offsets form the moiré pattern in the overlapped patterns in theoretically almost real-time, from step.1 to step.4 and from step.6 to step.8

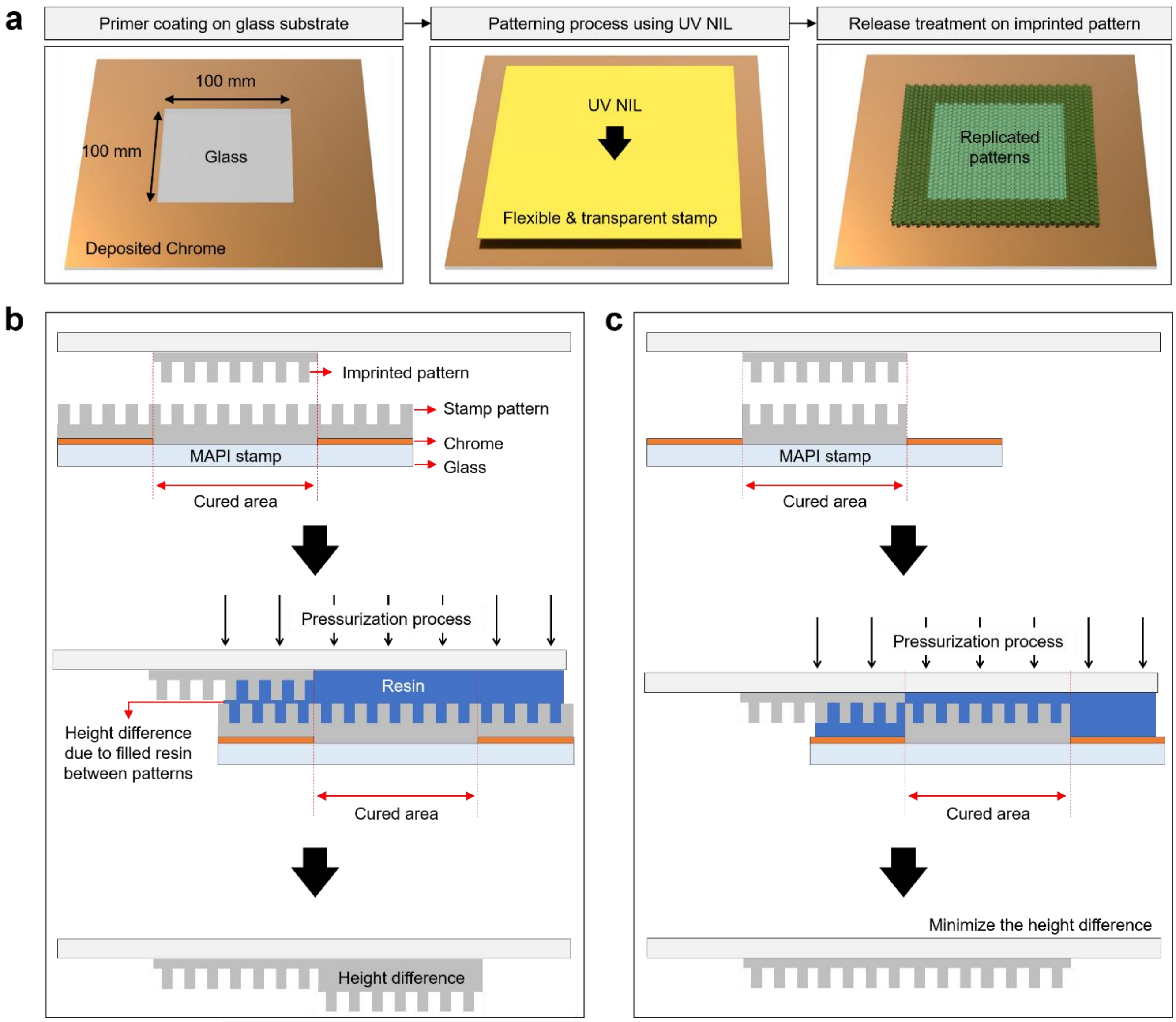

Supplementary Figure. 4 Schematic of the MAPI stamp fabrication. a-b Process flow of the MAPI stamp used in this study. **c** Process flow of the improved fabrication of the MAPI stamp.

It is important to consider the Δh when creating the multiple-stitched patterns with the MAPI process. Given the impact of the Δh on the overall quality of the stitched area, we suggest optimizing the fabrication process of the MAPI stamp to reduce or minimize this factor. The **supplementary Figure 4** can provide valuable insights and suggestions for achieving this optimization. Since the actual printed area was slightly larger than the transparent area in the chrome-coated shadow mask (**Supplementary Fig. 4a**), thus occurring an inevitable overlapped area between the already imprinted patterns and the MAPI stamp patterns during the sequential stitching process. The **supplementary Figure 4b** indicates that the presence of residual resin at the border of the overlapped region is the primary cause of the Δh observed in the stitched area. Consequently, we believe that further optimizing the fabrication process of the MAPI stamp (**Supplementary Fig. 4c**) to eliminate any size difference between the printed and transparent areas may help remove or minimize the residual resin in the overlapped region, which is the main cause of the Δh measured in the stitched area.

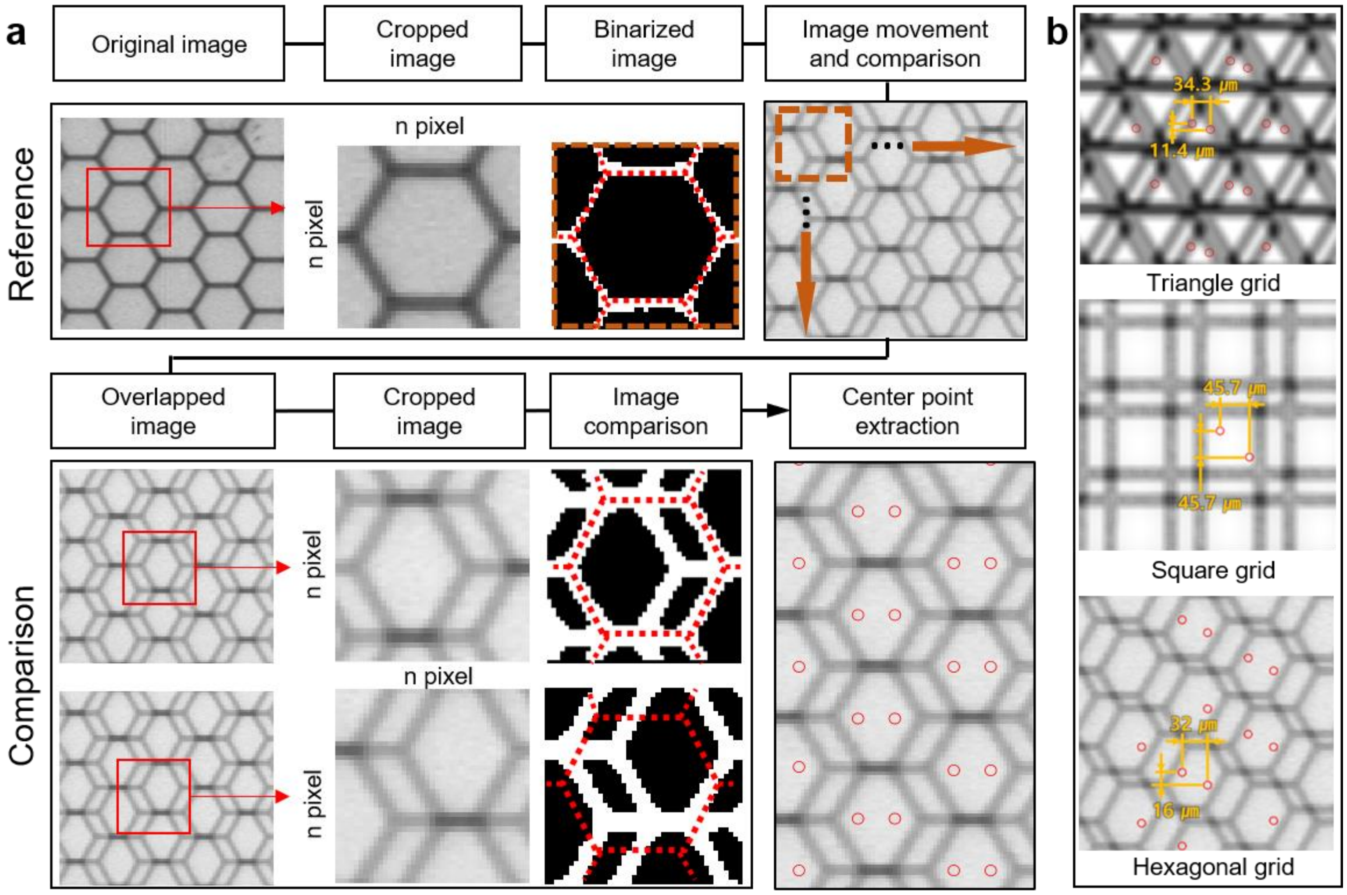

Supplementary Figure. 5 Compensation of translational offset by extracting center points with pixel shift analysis. a Flowchart on the extraction of the center point in the overlapped patterns. **b** Examples of the extraction of the center position in various periodic structures such as triangular, square, and hexagonal patterns.

Supplementary Figure 5 illustrates the functional block diagram of the vision-based pixel shift analysis to measure the translational offset of overlapped patterns. The pixel shift analysis involves a simple algorithm to extract the center coordinate of a unit cell in structured patterns (**Supplementary Fig. 5a**). Firstly, a unit cell image was cropped to an odd number of pixels from the non-overlapped optical image and set as a reference image. The reference image was then binarized to convert to the black inners and white outlines (**Supplementary Fig. 5a**). Next, the overlapped pattern image was also cropped and binarized with the same number of pixels for comparison with the reference image. By comparing the white outlines of these two images, the degree of superposition in terms of pixels was analyzed. Through this one-to-one comparison, the offset pixels to the center positions of each unit cell in the reference and overlapped images were counted and converted to the spatial displacement in the 2D directions (**Supplementary Fig. 5b**).

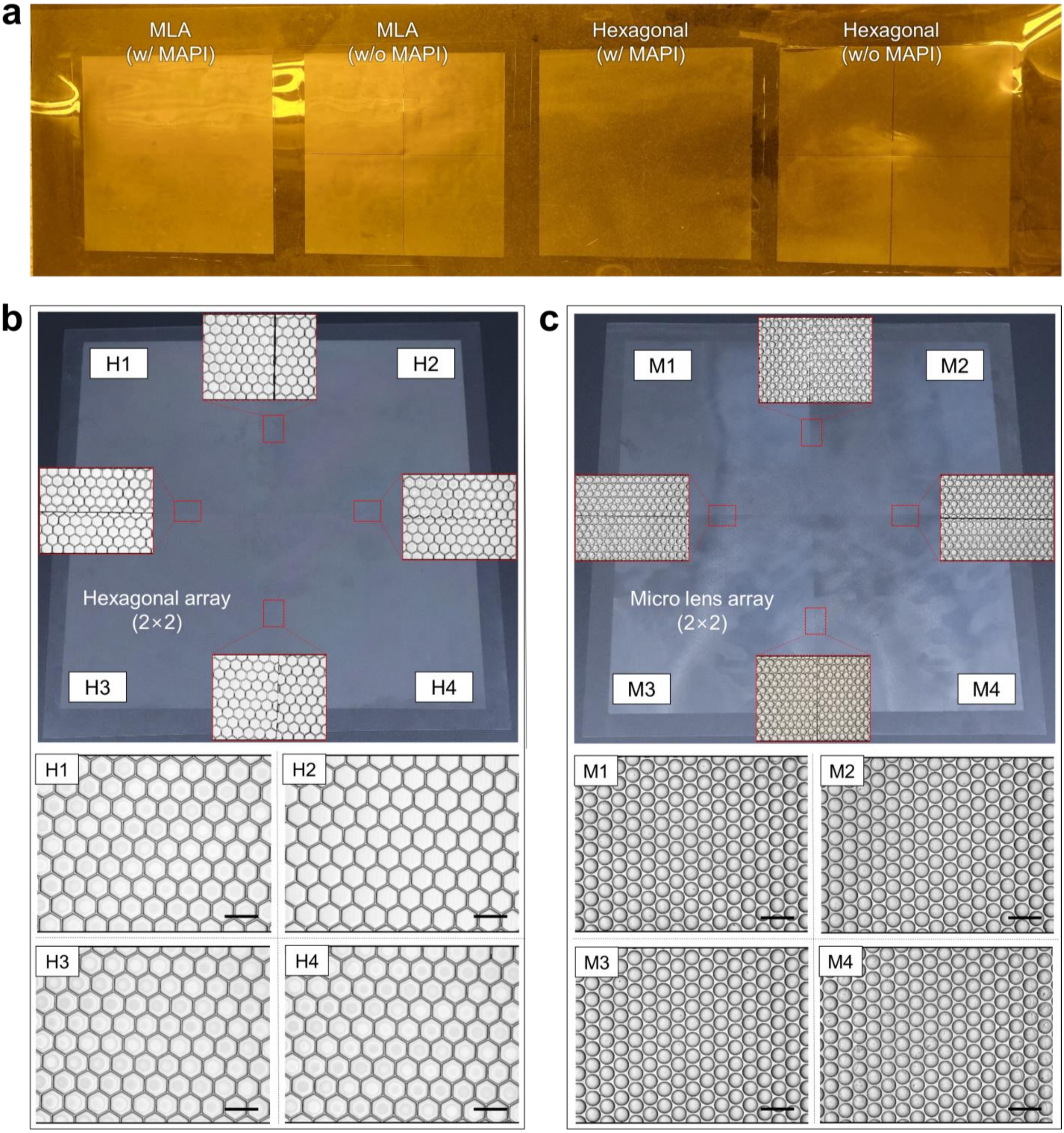

Supplementary Figure. 6 Large-area microstructured surfaces using step-and-repeat stitching based on the MAPI process. a Photographs of the multiple-stitched micropatterned surfaces using the MAPI and conventional manual process. 3D profiles of the MAPI-based stitched microstructured surfaces with quasi-seamlessness composed of **b** interconnected hexagonal microcavities and **c** MLA films. Scale bar: 100 μm.

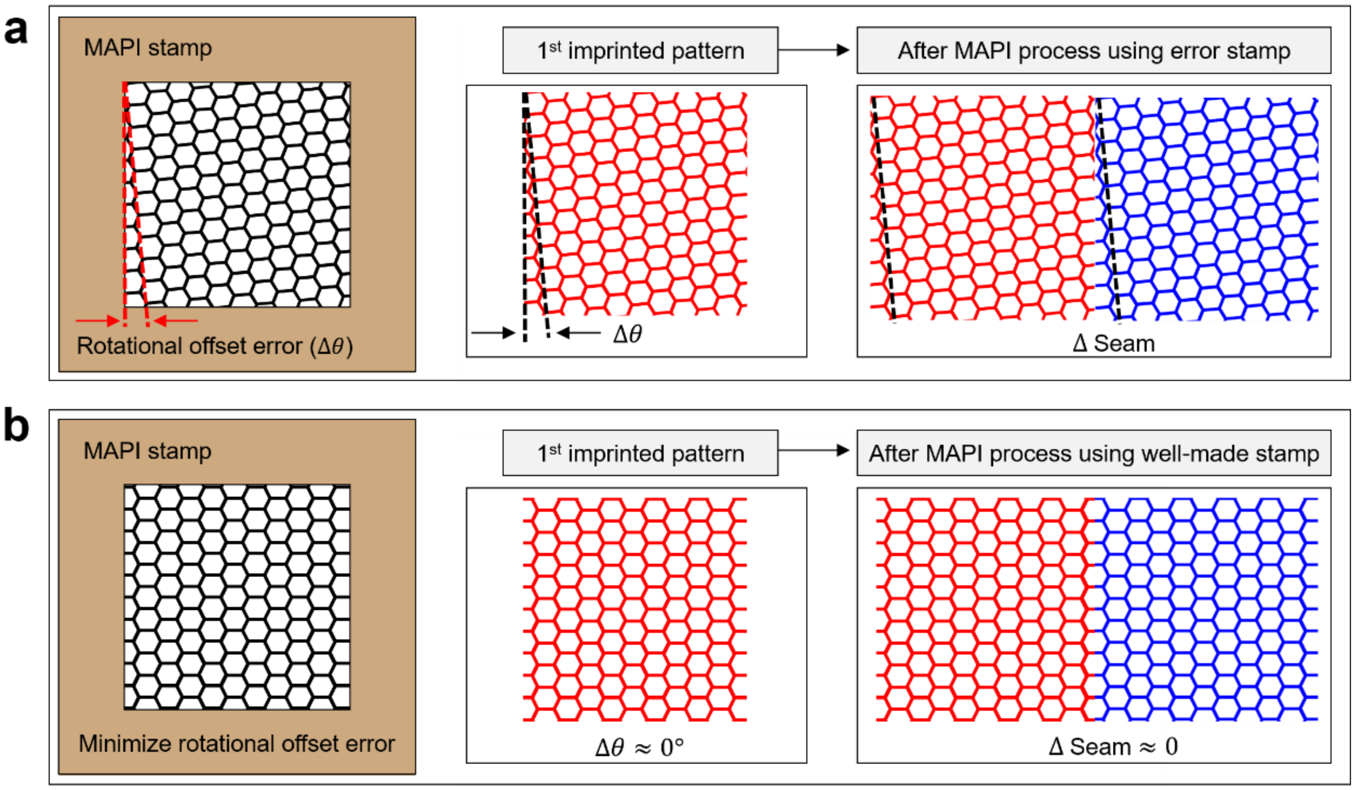

Supplementary Figure. 7 The translation offsets after the stitching process. a with and **b** without the rotational offset of the MAPI stamp.

During the fabrication process of the MAPI stamp, the resolution of mechanical rotation stage (around 0.1°) is attributed to the rotational offset between the transparent area of the chrome-coated shadow mask and mold patterns. For example, the rotational offset of 0.1° yields the translation offset of around 174 μm in the horizontal direction between both edges when considering the rectangular transparent area of 100 mm × 100 mm, as presented in the **supplementary Figure. 7**. We expect that a precise motorized rotation stage can help address the rotational offset in the fabrication of the MAPI stamp.

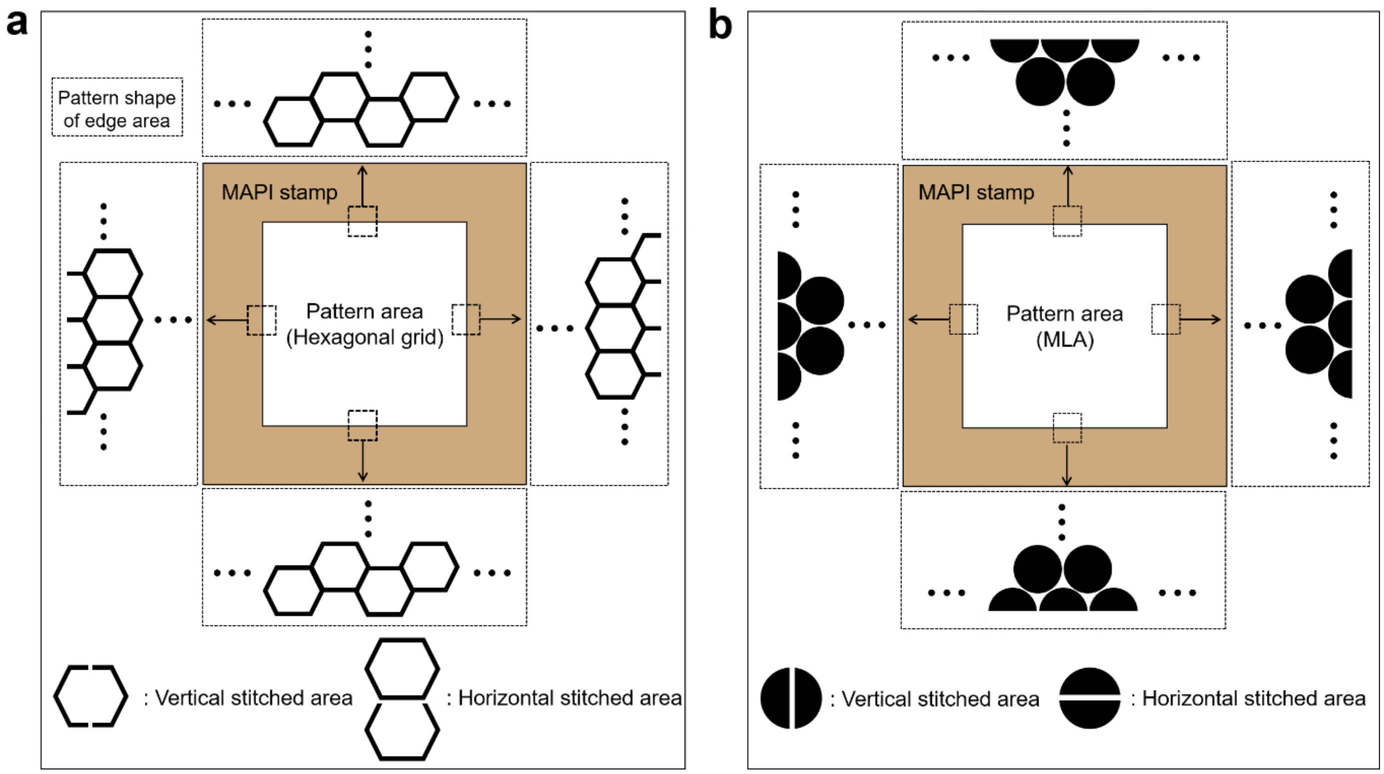

Supplementary Figure. 8 The design strategy of mold patterns in the MAPI stamp for minimizing the translational offsets. a Hexagonal grid pattern **b** Micro lens array pattern.

The transparent area in the shadow mask was not divided by an integer multiple of the unit cell size of the microstructures used in this study and this leads to discrepancy of the stitched patterns at the border lines (**Fig.4** in the main text). We suggest a potential solution to this discrepancy, which is to modify or optimize the mold pattern design to better align with the ending edge's shape of the microstructure unit at the border lines of the shadow mask. The schematic described in the **supplementary Figure. 8** in the revised manuscript may provide further guidance on how to achieve this optimization. By improving the alignment between the mold patterns and chrome-coated shadow mask, it may be possible to minimize the translational offsets and improve the overall quality and accuracy of the stitched patterns in the MAPI process.

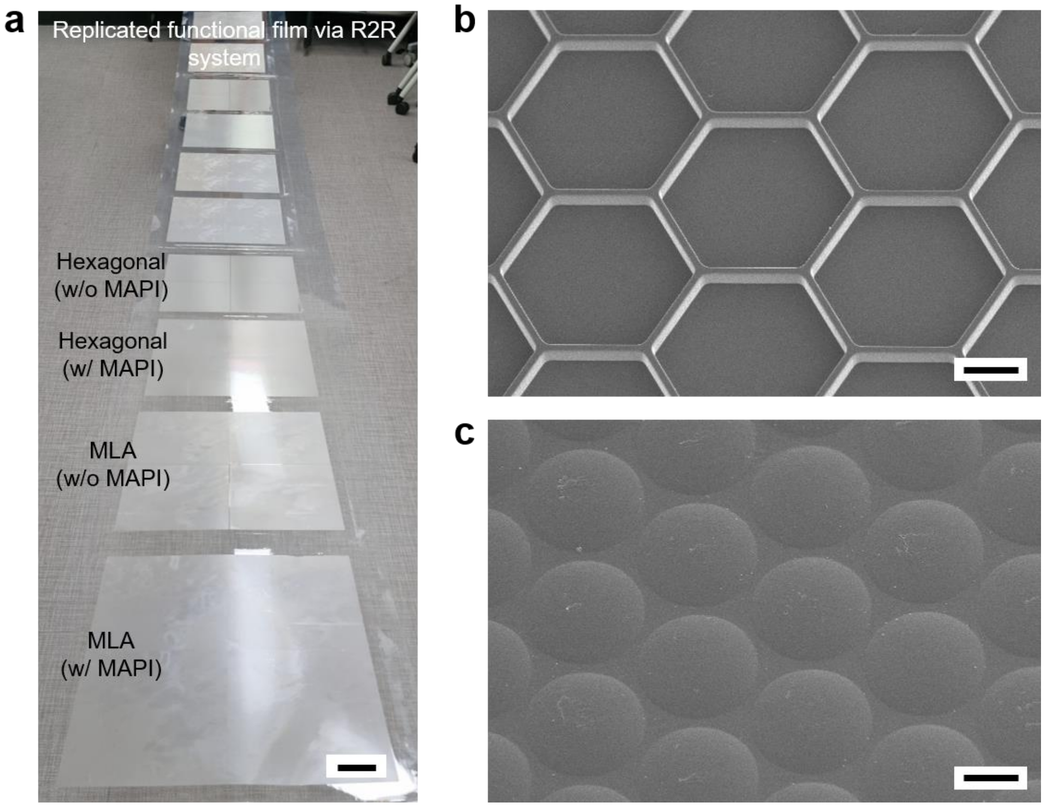

Supplementary Figure. 9 R2R-NIL replicated microstructured surfaces a Fabricated large area functional film using the continuous production system (Roll to roll). Scale bar: 20 mm. **b** SEM image of fabricated hexagonal grid pattern **c** SEM image of fabricated micro lens array pattern. Scale bar: 20 μm.

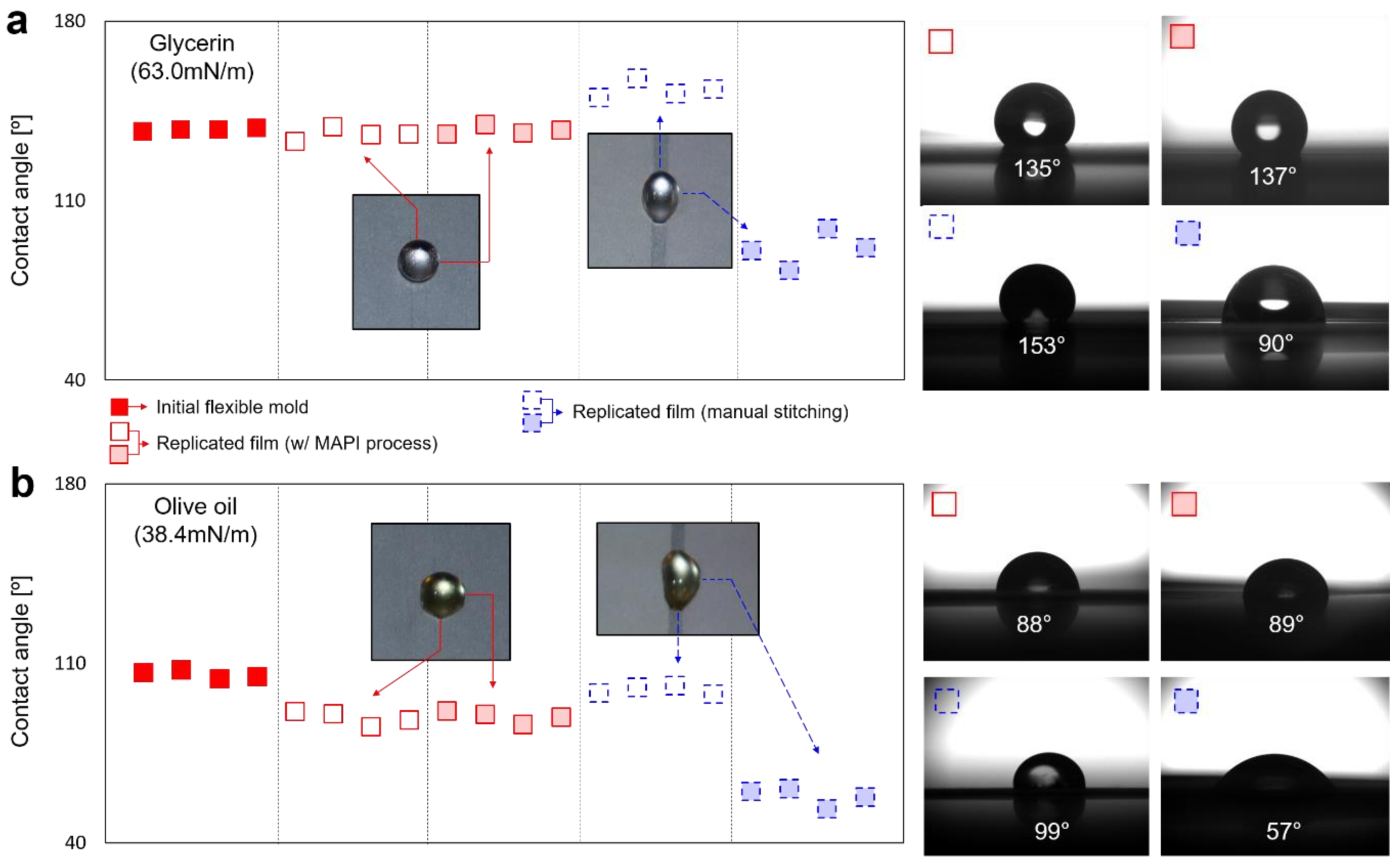

Supplementary Figure. 10 The measured contact angles on the initial mold and their replicated films fabricated using a multiple-stitched flexible mold a Glycerin **b** Olive oil

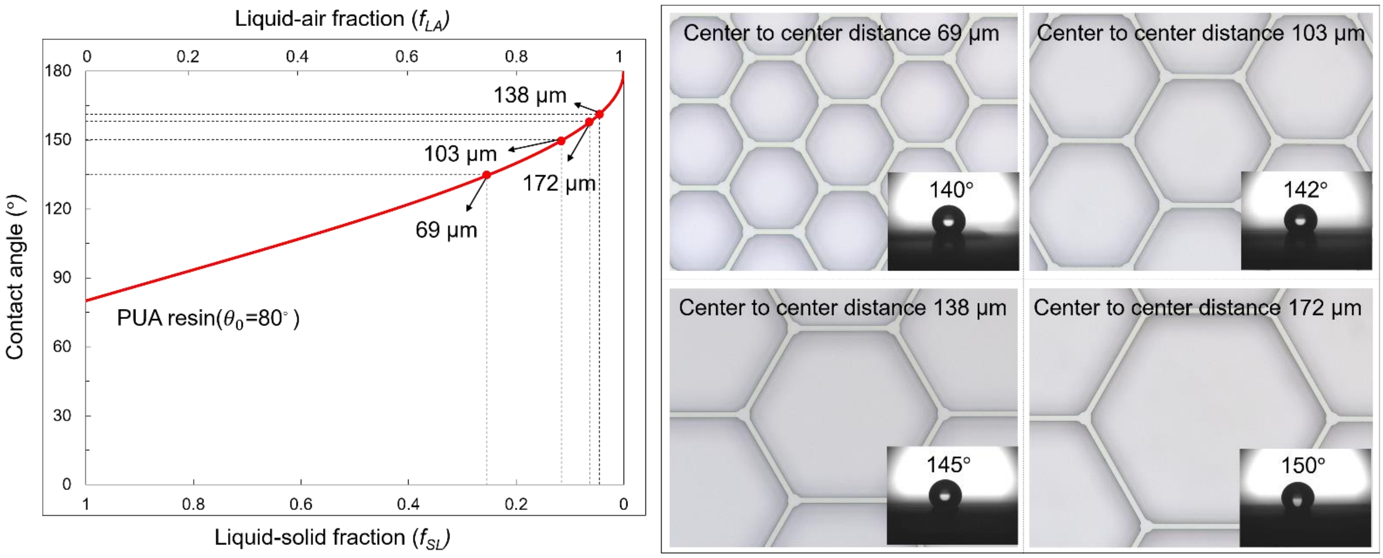

Supplementary Figure. 11 The improved hydrophobicity by controlling the geometrical parameter of the microstructured surfaces.

The wetting behavior of materials is mainly determined by the constituent material and the corresponding solid fraction, which is dependent on the geometry of surface patterns[4,5]. The micropatterned surfaces are not flat and have a certain surface roughness, and the contact angle can be determined using either the Wenzel model or the Cassie and Baxter model[4]. The Cassie-Baxter model assumes that when a droplet touches the micropatterned surface, the droplet is supported by the microstructures rather than being fully wetted inside the structures. The contact angle of liquid droplets can be determined in this model based on two factors: 1) the constituent material property, and 2) the geometrical property of microstructures, which is the liquid-solid fraction (or solid fraction), defined das the ratio of the area where the droplet touches the solid surface to the projected area. According to the Cassie-Baxter model, the wetting behavior of the microstructured surfaces can be controlled by adjusting the solid fraction, without changing the constituent material being used. In this study, we used an interconnected microcavity pattern with a width of 2 μm and center-to-center distance of 70 μm as a model structure to showcase the feasibility of the MAPI process. Although the primary objective of this study was not to demonstrate a superhydrophobic surface, but rather to showcase the potential of the MAPI process for creating large-area functional microstructured patterns, the hydrophobicity can still be improved by adjusting the geometrical parameter such as solid fraction in the microstructured surfaces. To support this claim, we conducted experimental measurements of the contact angle by reducing the solid fraction (increasing the center-to-center distance) with the same constituent material (MINS 311RM resin, Changsung sheet, Korea) used in this study. The results are presented in **Supplementary Figure. 11** and we confirmed that the contact angle above 150º can be achieved with a center-to-center distance of 172 μm in accordance with the Cassie-Baxter model.

Supplementary References

1. Lu, C. C., Lin, Y. C., Yeh, C. H., Suenaga, K. & Chiu, P. W., Twisting bilayer graphene superlattices. *ACS nano*, **7**, 2587-2594 (2013).
2. Han, Y., Gao, L., Zhou, J., Hou, Y., Cao, K., Duan, K. & Lu, Y., Deep elastic strain engineering of 2D materials and their twisted bilayers. *ACS Applied Materials &Interfaces*, **14**, 8655-8663 (2022).
3. Solis-Fernandez, P., & Ago, H., Machine Learning Determination of the Twist Angle of Bilayer Graphene by Raman Spectroscopy: Implications for van der Waals Heterostructures. *ACS Applied Nano Materials*, **5**, 1356-1366 (2022).
4. Kim, S., Kim, D. H., Choi, S. H., Kim, W. Y., Kwon, S., & Cho, Y. T. Effect of surface pattern morphology on inducing superhydrophobicity. *Applied Surface Science*, **513**, 145847 (2020).
5. Kim, D. H., Kim, S., Park, S. R., Fang, N. X., & Cho, Y. T. Shape-Deformed Mushroom-like Reentrant Structures for Robust Liquid-Repellent Surfaces. *ACS Applied Materials & Interfaces*, **13**, 33618-33626 (2021).
